# Association between triglyceride levels and hypertension in patients with coronary artery disease undergoing percutaneous coronary intervention: a retrospective cross-sectional study

**DOI:** 10.3389/fcvm.2025.1734117

**Published:** 2026-01-08

**Authors:** Zhanqun Gao, Yubo Gao, Chao Fang, Chen Zhao, Dirui Zhang, Wei Hao, Haibo Jia, Sining Hu, Bo Yu

**Affiliations:** 1 Department of Cardiology, The Second Affiliated Hospital of Harbin Medical University, Harbin, China; 2 Key Laboratory of Myocardial Ischemia, Chinese Ministry of Education, Harbin, China; 3 Department of Cardiovascular Medicine, General Hospital of Hunan Medical College, Hunan, China

**Keywords:** triglycerides, coronary artery disease, hypertension, percutaneous coronary intervention, J-shaped association

## Abstract

**Background:**

This study aimed to investigate the association between serum triglyceride (TG) levels and the presence of hypertension in patients with coronary artery disease (CAD) and hypertension undergoing percutaneous coronary intervention (PCI).

**Methods:**

This retrospective study included 3,150 patients diagnossed with CAD who underwent PCI after coronary angiography (CAG) at the Department of Cardiology, The Second Affiliated Hospital of Harbin Medical University, between September 8, 2019, and February 27, 2022. Serum TG levels were measured within 24 h of admission, and covariates, including demographic data, clinical characteristics, and serum biomarkers, were collected. Multivariate logistic regression models were used to analyze the association between TG levels and hypertension.

**Results:**

A total of 36.8% (1,159/3,150) were ultimately included in the analysis, with 27.5% (319/1,159) presenting acute myocardial infarction (AMI). After adjusting for multiple confounding factors, TG levels were found to be independently associated with the presence of hypertension [odds ratio [OR] = 1.23, 95% confidence interval [CI]: 1.03–1.47]. When grouped by TG level quartiles, compared with the Q1 group, Q2, Q3, and Q4 showed significantly higher risks of hypertension in patients with CAD [OR, 95% CI: 1.98 (1.39–2.81), 1.80 (1.25–2.58), and 2.61 (1.68–4.05), respectively]. Furthermore, an additional analysis revealed a nonlinear relationship between TG and hypertension, characterized by a pronounced saturation effect which was particularly pronounced in patients with ejection fraction <50%.

**Conclusion:**

This study is the first to demonstrate a J-shaped association between TG level and hypertension presence in patients with CAD after PCI, with an inflection point of 3.86 mmol/L. This association was more pronounced in patients with reduced EF, suggesting that in patients with cardiac dysfunction, there is a stronger association between TG levels and hypertension, which may warrant further investigation into the clinical benefits of TG management in this subgroup.3

## 1 Background

Coronary artery disease (CAD) is the leading cause of morbidity and mortality worldwide. Percutaneous coronary intervention (PCI) is a critical revascularization strategy to effectively restore coronary blood flow (1). Hypertension is the most common and modifiable risk factor for cardiovascular disease; it frequently coexists with CAD and significantly increases the risk of adverse outcomes (2). Despite significant advances in revascularization strategies and pharmacotherapy in recent years, the incidence of adverse cardiovascular events remains high among patients after PCI, highlighting the urgent need for more refined risk stratification and optimized management strategies for this population.

Dyslipidemia plays a pivotal role in the development and progression of CAD. Elevated triglyceride (TG) levels are closely associated with atherosclerotic progression and contribute to the development and progression of hypertension through mechanisms such as impaired endothelial function and enhanced inflammatory responses (3). Currently, low-density lipoprotein cholesterol (LDL-C) is a core intervention target in atherosclerotic cardiovascular disease (4). However, the independent impact of TG on the presence of hypertension in patients with PCI-treated CAD remains unclear, suggesting the complexity and multidimensional effects of TG on cardiovascular pathophysiology (5). Furthermore, whether cardiac dysfunction modifies this association remains unclear. Therefore, this study aimed to examine the cross-sectional association between TG levels and the prevalence of hypertension in patients with PCI-treated CAD, with a specific focus on the effect-modifying role of left ventricular ejection fraction (EF).

Coronary artery disease accounts for 30% of disease-related mortality (6) and remains a leading cause of morbidity and mortality globally (7), posing a significant public health challenge. Hypertension affects approximately 31% of adults worldwide, and this figure is expected to reach 60% by 2025 (8). Hypertension, which is characterized by elevated blood pressure, has an uncertain etiology and may be associated with factors such as age, genetics, and lifestyle (9). It serves as a primary risk factor for cardiovascular events such as stroke and myocardial infarction, while substantially increasing the socioeconomic burden of healthcare (10). Notably, patients with CAD and hypertension face persistent residual risks, even after revascularization. Thus, the early identification of controllable risk factors (such as TG levels) is crucial for improving long-term prognosis.

Persistently elevated TG levels contribute to hypertension through multiple pathways, including the induction of oxidative stress, impairment of endothelium-dependent vasodilation, and promotion of vascular remodeling (11). Additionally, elevated TG levels promote the development of atherosclerosis, leading to thickened and roughened arterial walls, narrowed lumens, impaired cardiac blood supply, enhanced platelet aggregation, and thrombosis, thereby exacerbating CAD (12). Although the relationship between TG and cardiovascular disease has received extensive attention, its specific role in patients with hypertension who have undergone PCI, particularly its interaction with cardiac function, warrants further investigation.

This study aimed to explore the association between TG and hypertension risk in patients with CAD who have undergone PCI, with a focus on evaluating the potential effect-modifying role of EF. This study aimed to provide new evidence-based guidelines for lipid management and prevention of hypertension in this population.

## 2 Methods

### 2.1 Study population

**Figure 1:**
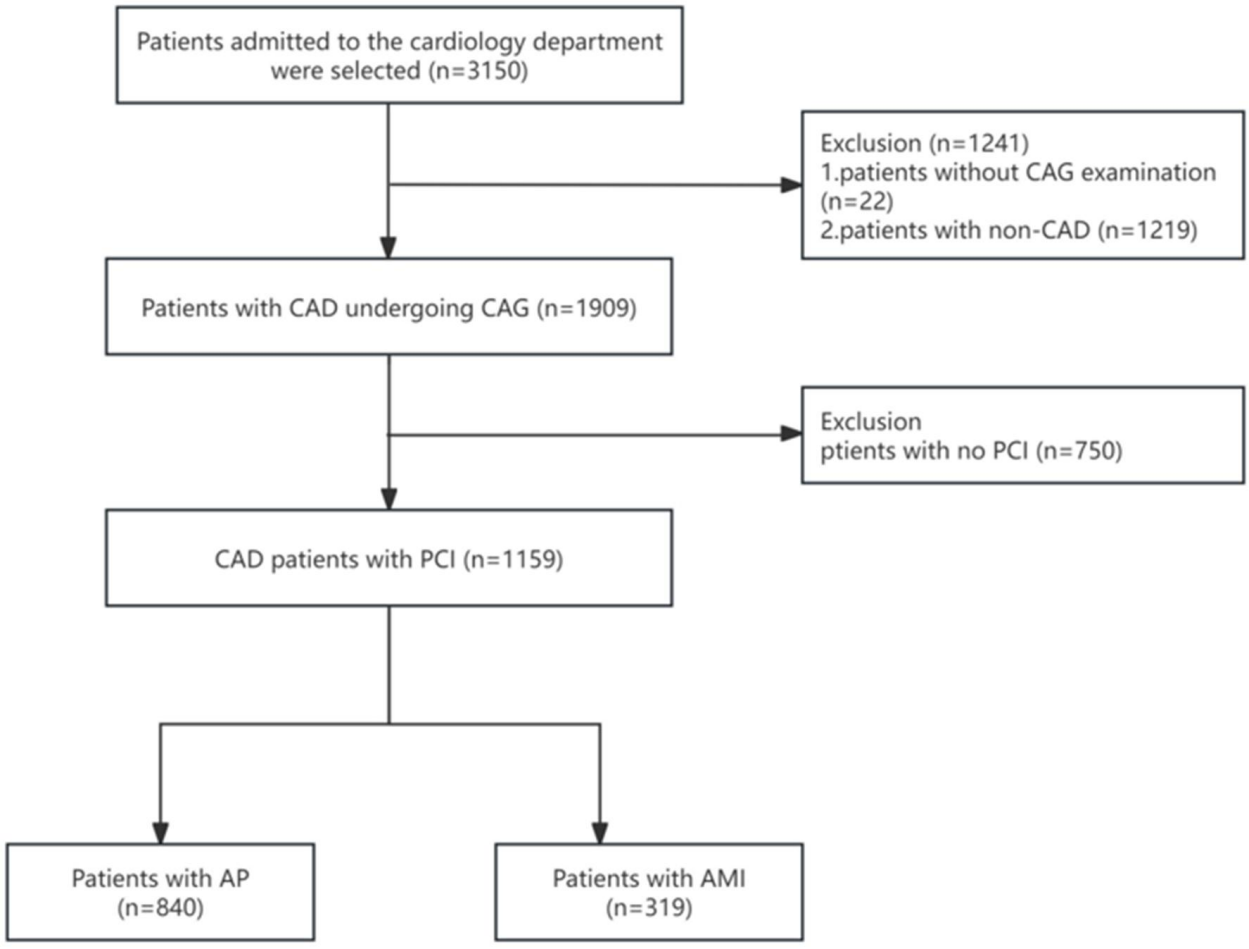
Study design and participant flow diagram for the present study. AMI, acute myocardial infarction; AP, angina pectoris; CAD, coronary artery disease; CAG, coronary angiography; PCI, percutaneous coronary intervention.

This retrospective, single-center, cross-sectional analysis consecutively enrolled adult patients aged 21–90 years with suspected CAD who underwent coronary angiography (CAG)-based examination and PCI at the Department of Cardiology, The Second Affiliated Hospital of Harbin Medical University, between September 8, 2019, and February 27, 2022. The exclusion criteria were as follows: ① Patients who did not undergo CAG; ② Patients with no significant CAD on CAG; and, ③ Patients who did not undergo PCI. Ultimately, 1,159 patients were included in the analysis (Figure 1).

### 2.2 Data collection and measurement

Random blood sugar samples were collected within 24 h of admission from all subjects. The serum was separated by centrifugation at 3,000 rpm for 10 min and stored at −80 °C for the subsequent analysis. Triglyceride concentrations were quantified using the glycerophosphate oxidase-peroxidase (GPOPAP) method with a commercial kit (Catalog No.: CH0101151) from Beijing Jiuqiang Biotechnology Co., Ltd. (Roche Diagnostics, Basel, Switzerland). The intra- and inter-assay coefficients of variation (CV) for the internal quality control materials in this laboratory were both less than 5%, meeting the quality control standards. And AMI categories, with the latter identified by typical symptoms, ST-segment changes on electrocardiogram (13), and elevated cardiac enzymes (14). Hypertension was defined as systolic blood pressure ≥140 mmHg and/or diastolic blood pressure ≥90 mmHg, or current use of antihypertensive medication (15). Left ventricular ejection fraction (EF) was assessed using transthoracic echocardiography during the index hospitalization. The measurement was performed by experienced sonographers in accordance with the current guidelines of the American Society of Echocardiography, and the biplane Simpson's method of discs was the preferred technique for calculation.

Demographic characteristics, clinical history (including diabetes, stroke, smoking, and alcohol consumption), ancillary test results, and laboratory parameters were collected. These included EF and levels of creatinine (Cr), random blood sugar (RBS), C-reactive protein (CRP), total cholesterol (TC), high-density lipoprotein cholesterol (HDL-C), LDL-C, glycated hemoglobin (HbA1c), etc.

### 2.3 Statistical methods

Precise 95% confidence intervals (CI) were calculated for all the diagnostic performance metrics. Continuous variables were presented as mean ± standard deviation, whereas categorical variables were described as frequency (percentage, %). Group comparisons employed t-tests or *χ*^2^ tests. Univariate and multivariate logistic regression analyses were performed to assess the association between TG levels and hypertension. The odds ratios (OR) and 95% CIs were calculated. Trend tests were performed by TG level quartile grouping. Restricted cubic spline (RCS) models were used to fit the nonlinear relationship between TG and hypertension using an inflection point analysis. Subgroup analyses were stratified by age, sex, HbA1c level, and EF, and interactions were tested. All analyses were performed using the statistical software package R (http://www.R-project.org; R Foundation) and Free Statistics software version 1.9.1. Statistical significance was set at *P* < 0.05.

## 3 Results

### 3.1 General description

Patients with CAD undergoing PCI were divided into two groups based on their hypertension status: the hypertension group (56.9%, 660/1,159) and the non-hypertension group (43.1%, 499/1,159). In the hypertensive group, the proportion of patients with angina was significantly higher than that of patients with AMI (*P* < 0.05), and the proportions of patients without diabetes and stroke were also significantly higher than those of patients with diabetes and stroke (*P* < 0.001). Additionally, the proportion of female patients in the hypertensive group was significantly higher than that in the non-hypertensive group (*P* < 0.05), suggesting that sex may be an important factor influencing the development of TG-induced hypertension. Compared with the non-hypertensive group, patients in the hypertensive group had significantly higher TG levels (2.0 ± 1.3 mmol/L vs. 1.8 ± 1.3  mmol/L; *P* < 0.01) and were older (60.2 ± 9.7 years vs. 57.6 ± 10.0 years; *P* < 0.001), indicating that dyslipidemia and advancing age are closely associated with hypertension development. Additionally, significant differences in Cr, HDL-C, and HbA1c levels were observed between the two groups, suggesting that renal function, lipid metabolism, and glycemic control may play synergistic roles in the pathogenesis of hypertension (Table 1).

**Table 1:** Characteristics of the study participants.

Variables	Total (*n* = 1,159)	Non-hypertension group (*n* = 499)	Hypertension group (*n* = 660)	*P*
AMI, *n* (%)				0.027
No	840 (72.5)	345 (69.1)	495 (75)	
Yes	319 (27.5)	154 (30.9)	165 (25)	
Age, Mean ± SD	59.1 ± 9.9	57.6 ± 10.0	60.2 ± 9.7	<0.001
Sex, *n* (%)				0.017
Male	812 (70.1)	368 (73.7)	444 (67.3)	
Female	347 (29.9)	131 (26.3)	216 (32.7)	
Diabetes, *n* (%)				<0.001
No	850 (73.3)	403 (80.8)	447 (67.7)	
Yes	309 (26.7)	96 (19.2)	213 (32.3)	
Stroke, *n* (%)				< 0.001
No	1,008 (87.0)	460 (92.2)	548 (83)	
Yes	151 (13.0)	39 (7.8)	112 (17)	
Smoking, *n* (%)				0.072
Never	586 (50.6)	245 (49.1)	341 (51.7)	
Former	424 (36.6)	199 (39.9)	225 (34.1)	
Current	149 (12.9)	55 (11)	94 (14.2)	
Drinking, *n* (%)				0.742
Never	892 (77.0)	389 (78)	503 (76.2)	
Former	209 (18.0)	85 (17)	124 (18.8)	
Current	58 (5.0)	25 (5)	33 (5)	
EF, Mean ± SD (%)	59.9 ± 6.9	59.5 ± 7.6	60.2 ± 6.3	0.104
Cr, Mean ± SD (umol/L)	80.4 ± 31.3	77.1 ± 18.1	82.9 ± 38.2	0.002
RBS, Mean ± SD (mmol/L)	6.7 ± 2.6	6.6 ± 2.6	6.8 ± 2.6	0.143
CRP, Mean ± SD (mg/L)	3.8 ± 3.8	3.7 ± 3.8	3.9 ± 3.8	0.264
T.CH, Mean ± SD (mmol/L)	4.4 ± 1.1	4.4 ± 1.1	4.4 ± 1.0	0.717
TG, Mean ± SD (mmol/L)	1.9 ± 1.3	1.8 ± 1.3	2.0 ± 1.3	0.002
HDL, Mean ± SD (mmol/L)	1.0 ± 0.2	1.1 ± 0.3	1.0 ± 0.2	0.012
LDL, Mean ± SD (mmol/L)	2.8 ± 0.9	2.8 ± 0.9	2.7 ± 0.9	0.115
HbAlc, Mean ± SD (%)	6.5 ± 1.4	6.4 ± 1.4	6.6 ± 1.3	0.003

Data are presented as *n* (%) or Mean ± SD. *P*-values were derived from independent t-tests for continuous variables and *χ*^2^ tests for categorical variables. AMI, acute myocardial infarction; Cr, Creatinine; RBS, random blood sugar; CRP, c-reactive protein; T.CH, total cholesterol; TG, Triglycerides; HDL, high-density lipoprotein cholesterol; LDL, low-density lipoprotein cholesterol; HbA1c, glycated hemoglobin.

### 3.2 Univariate analysis

Univariate analysis revealed significant positive correlations between AMI; age; sex; diabetes; stroke; serum Cr, TG, HDL-C, and HbA1c levels; and, hypertension risk (*P* < 0.05). Significant differences in age, sex, diabetes, and stroke were observed between the two groups. These factors are not only associated with hypertension onset but may also influence CAD progression and post-intervention outcomes through multiple pathophysiological pathways. This analysis provides a foundation for subsequent multivariate adjustments (Table 2).

**Table 2:** Univariate logistic regression analysis of factors associated with hypertension.

Variable	OR (95% CI)	*P*-value
AMI	0.75 (0.58–0.97)	0.027
Age	1.03 (1.01–1.04)	<0.001
Female	1.37 (1.06–1.77)	0.017
Diabetes	2 (1.52–2.64)	<0.001
Stroke	2.41 (1.64–3.54)	<0.001
EF	1.01 (1–1.03)	0.105
Cr	1.01 (1–1.02)	0.001
RBS	1.04 (0.99–1.08)	0.144
CRP	1.02 (0.99–1.05)	0.264
T.CH	0.98 (0.88–1.09)	0.716
TG	1.18 (1.06–1.31)	0.002
HDL	0.54 (0.34–0.88)	0.013
LDL	0.9 (0.79–1.03)	0.115
HbAlc	1.15 (1.05–1.26)	0.003

Data are presented as Odds Ratio (OR) with 95% Confidence Interval. AMI, acute myocardial infarction; CI, confidence interval; Cr, creatinine; CRP, C-reactive protein; EF, ejection fraction; HbA1c, glycated hemoglobin; HDL, high-density lipoprotein cholesterol; LDL, low-density lipoprotein cholesterol; OR, odds ratio; RBS, random blood sugar; T.CH, total cholesterol; TG, triglycerides.

### 3.3 Association between triglyceride level quartiles and hypertension

Multivariate logistic regression analysis (*n* = 1,159) examined the association between the TG level quartiles and hypertension (Table 3). In the unadjusted model, TG levels showed a significant positive correlation with hypertension (OR = 1.18, 95% CI: 1.06–1.31). After adjusting for age and sex in Model I, the association strengthened (OR = 1.22, 95% CI: 1.10–1.36), indicating that demographic factors are important confounders. Model II, further adjusted for diabetes, alcohol consumption, smoking, and stroke history, yielded results similar to Model I (OR = 1.22, 95% CI: 1.09–1.35), suggesting these clinical and behavioral factors had limited impact on the association. Model III, which further adjusted for Cr, RBS, CRP,TC, HDL-C, LDL-C, and HbA1c levels based on Model II, still showed a significant association between TG level and hypertension (OR = 1.23, 95% CI: 1.03–1.47), indicating that TG level is an independent risk factor for hypertension. To further validate the robustness of the results, a sensitivity analysis was conducted by grouping TG levels into quartiles. The results showed a positive correlation between TG levels and the risk of hypertension. Compared with the lowest quartile (Q1), the highest quartile (Q4) showed a significantly increased risk of hypertension (OR = 2.61, 95% CI: 1.68–4.05), further supporting the important role of TG in the development of hypertension.

**Table 3:** Regression analysis between TG quartiles and hypertension.

Variable	Crude model		Model 1		Model 2		Model 3	
	OR (95%CI)	*P* value	OR (95%CI)	*P* value	OR (95%CI)	*P* value	OR (95%CI)	*P* value
TG	1.18 (1.06–1.31)	0.002	1.22 (1.10–1.36)	<0.001	1.22 (1.09–1.35)	<0.001	1.23 (1.03–1.47)	0.02
TG (quartile)								
Q1	Ref		Ref		Ref		Ref	
Q2	1.93 (1.39–2.69)	<0.001	1.95 (1.39–2.72)	<0.001	1.98 (1.41–2.79)	<0.001	1.98 (1.39–2.81)	<0.001
Q3	1.73 (1.24–2.4)	0.001	1.8 (1.29–2.51)	0.001	1.77 (1.26–2.49)	0.001	1.8 (1.25–2.58)	0.002
Q4	2.4 (1.72–3.36)	<0.001	2.69 (1.91–3.8)	<0.001	2.62 (1.84–3.73)	<0.001	2.61 (1.68–4.05)	<0.001
P for trend	1.29 (1.16–1.43)	<0.001	1.34 (1.20–1.49)	<0.001	1.32 (1.18–1.48)	<0.001	1.33 (1.16–1.52)	<0.001

Data are presented as Odds Ratio (OR) with 95% Confidence Interval (CI). Original Model: No factors adjusted. Model 1: Adjusted for age and gender. Model 2: Adjusted for diabetes, alcohol consumption, smoking, and stroke on top of Model 1. Model 3: Adjusted for Cr, random RBS, CRP, TC, HDL, LDL, and HbA1c based on Model 2. Cr, creatinine; CRP, C-reactive protein; HbA1c, glycated hemoglobin; HDL, high-density lipoprotein cholesterol; LDL, low-density lipoprotein cholesterol; RBS, random blood sugar; TC, total cholesterol.

### 3.4 Saturation effect analysis of TG and hypertension

**Figure 2:**
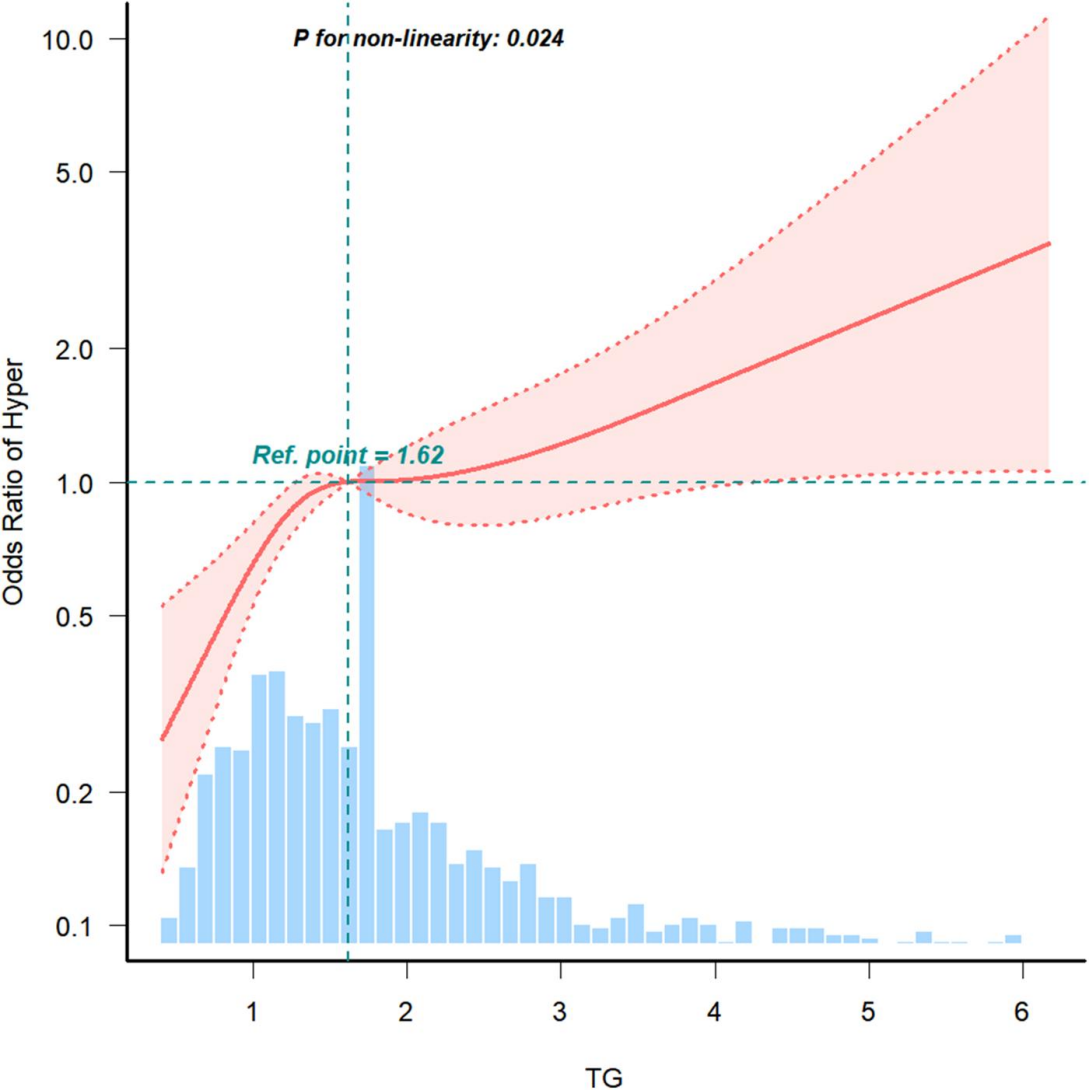
The fitted curve. The fitted curve shows a J-shaped relationship between TG level and CAD with hypertension underwent PCI. The solid line indicates the adjusted hazard ratio and the dashed lines the 95% confidence interval bands. CAD, coronary artery disease; PCI, percutaneous coronary intervention; TG, triglyceride.

Restricted cubic spline analysis (Figure 2) revealed a nonlinear relationship between the TG level and hypertension after adjusting for sex, age, diabetes, alcohol consumption, smoking, stroke, AMI, and Cr, RBS, CRP, TC, HDL-C, LDL-C, and HbA1c levels (*P* = 0.024). This manifested as a gradually decreasing curved slope with increasing TG levels, indicating a saturation effect. Breakpoint analysis further validated this phenomenon: when TG levels were below 3.863 mmol/L, the risk of hypertension increased with increasing TG levels. However, when TG levels exceeded 3.863 mmol/L, the hypertension risk no longer showed a significant increase (Table 4), suggesting that above this threshold, other factors may become more dominant, or the effect of TG may reach a plateau. This finding may have important implications for setting clinical intervention targets.

**Table 4:** Subgroup analysis.

Subgroup	n.total	n.event_%	crude.OR (95% CI)	crude.P_value	adj. OR (95%CI)	adj.P_value	P for interaction
Age							
<60 years	566	297 (52.5)	1.18 (1.04–1.35)	0.009	1.39 (1.08–1.8)	0.012	0.704
≥60 years	593	363 (61.2)	1.26 (1.04–1.52)	0.018	1.13 (0.87–1.48)	0.364	
HbAlc							
<6.0%	465	241 (51.8)	1.26 (1.03–1.56)	0.026	1.3 (0.98–1.73)	0.069	0.386
≥6.0%	694	419 (60.4)	1.11 (0.99–1.25)	0.073	1.14 (0.91–1.44)	0.263	
EF							
<50%	91	44 (48.4)	2.01 (1–4.06)	0.051	4.28 (1.47–12.43)	0.008	0.043
≥50%	1,068	616 (57.7)	1.15 (1.04–1.28)	0.007	1.13 (0.95–1.34)	0.165	
Sex							
male	812	444 (54.7)	1.21 (1.06–1.37)	0.005	1.31 (1.03–1.66)	0.026	0.258
female	347	216 (62.2)	1.1 (0.93–1.31)	0.26	1.08 (0.84–1.4)	0.529	

Data are presented as OR with 95% CI unless otherwise specified. Model descriptions: Crude model: Unadjusted. Adjusted model: Multivariable model. CI, confidence interval; EF, ejection fraction; HbA1c, glycated hemoglobin; OR, odds ratio.

### 3.5 Subgroup analysis

**Figure 3:**
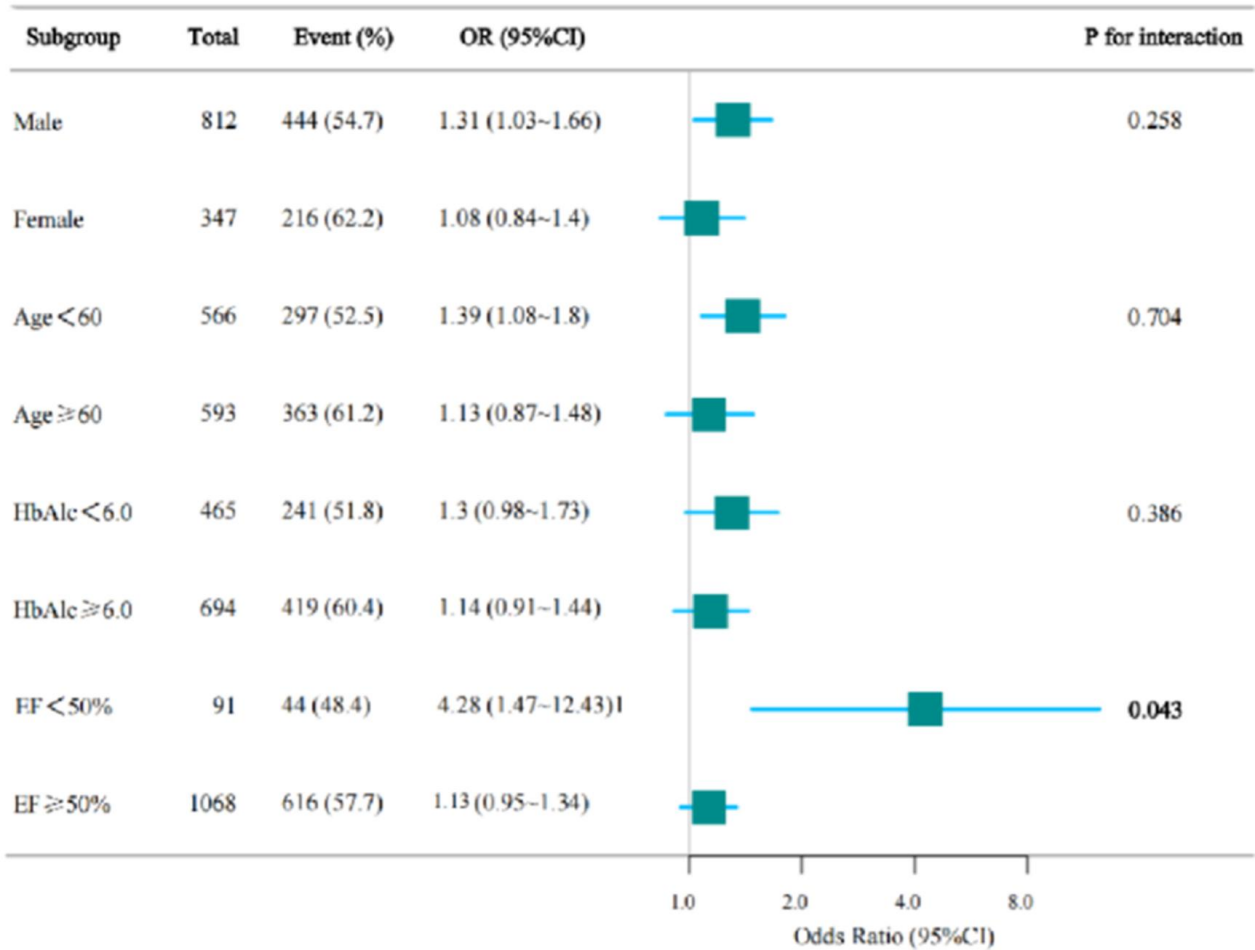
Forest plot. CI, confidence interval; EF, ejection fraction; HbA1c, glycated hemoglobin; OR, odds ratio.

To further explore the influence of other risk factors on the association between TG level and hypertension, subgroup analyses were conducted based on sex (male/female), age (<60/≥60 years), HbA1c (<6.0%/≥6.0%), and EF (<50%/≥50%). The results showed the strongest association between TG level and hypertension in the <50% EF subgroup (interaction, *P* < 0.05), suggesting that cardiac dysfunction may amplify the effect of TG on hypertension. No significant interactions were observed in the sex, HbA1c, or age subgroups, indicating that although these factors independently influence hypertension, they do not substantially alter the relationship between TG level and hypertension. These findings highlight the importance of strict TG level control in patients with heart failure. (Table 4 and Figure 3).

## 4 Discussion

This retrospective cross-sectional study, for the first time, reveals an independent J-shaped association between TG levels and hypertension in a population of patients with CAD undergoing PCI, with an inflection point at approximately 3.86 mmol/L. More importantly, we identified left ventricular EF as a significant effect modifier of this relationship; the positive association between TG and hypertension was markedly amplified in patients with cardiac dysfunction (EF < 50%), as evidenced by an OR of 4.28. These findings suggest that TG management may hold clinical importance in the secondary prevention of CAD that extends beyond current understanding, particularly in patients with concomitant impaired cardiac function.

### 4.1 The J-shaped association and the saturation effect

The nonlinear relationship observed in our study offers a fresh perspective on the complex role of TG in cardiovascular disease. Below the inflection point of 3.86 mmol/L, TG levels exhibited a positive correlation with the risk of hypertension, which aligns with the conclusions of numerous previous studies. The increased risk in this range is likely driven by several mechanisms: elevated TG levels induce systemic inflammatory responses and oxidative stress, impair nitric oxide-mediated endothelium-dependent vasodilation, and promote vascular remodeling; This pathophysiological cascade, encompassing systemic inflammation, oxidative stress, and impaired vasodilation, provides a mechanistic foundation for the observed association (16), these processes collectively contribute to increased peripheral vascular resistance and blood pressure (11, 17). However, when the TG concentration exceeded this threshold of 3.86 mmol/L, the association with hypertension risk demonstrated a saturation effect, wherein the risk no longer increased significantly with further rises in concentration. This J-shaped pattern contrasts with the linear associations often reported in general populations, underscoring the unique pathophysiological state of post-PCI patients (18),The non-linear, J-shaped relationship we identified between triglyceride levels and hypertension is consistent with the complex dynamics observed for other established cardiovascular risk factors. Notably, a dose-response meta-analysis has revealed a similar non-linear association between resting heart rate and the incidence of hypertension, suggesting that such threshold effects may be a fundamental characteristic in the pathophysiology of cardiovascular disease (19). A plausible explanation for this phenomenon is that at very high TG concentrations, the physical size of lipoprotein particles increases substantially (20), thereby reducing their ability to penetrate the arterial intima and cause direct damage to endothelial cells. This leads to a plateau in their direct atherogenic effects.

This leads to a plateau in their direct atherogenic effects. Notably, the inflection point identified in our study (3.86 mmol/L or ∼340 mg/dL) is substantially higher than the levels associated with increased cardiovascular risk in general populations (e.g., >89 mg/dL, as reported in recent studies). This discrepancy may be explained by the distinct clinical profile of our cohort, which comprised patients undergoing elective PCI, predominantly without acute myocardial infarction.Thus, our findings underscore the limitations of applying the traditional cardiovascular risk stratification system to this specific patient population (21). In this high-risk setting, it is highly probable that most patients were receiving guideline-directed medical therapy, including statins. Therefore, the measured TG levels represent on-treatment or residual concentrations (22). We hypothesize that in such a pharmacologically managed population, the threshold at which TG exerts a discernible effect on hypertension risk is shifted upward, possibly because its pathophysiological impact must be substantial enough to manifest against the backdrop of intensive secondary prevention. This observation highlights the context-dependent nature of TG-associated risk and underscores the need for population-specific risk stratification thresholds rather than a uniform approach. The non-linear, J-shaped relationship we identified between triglyceride levels and hypertension is consistent with the complex dynamics observed for other established cardiovascular risk factors (23).

### 4.2 TG, hypertension, and the amplifying effect of cardiac dysfunction

One of the most instructive findings of our study is the clarification of the significant amplifying effect of cardiac dysfunction (EF < 50%) on the association between TG and hypertension. This phenomenon may stem from multifaceted pathophysiological interactions. In the setting of heart failure, pre-existing neurohormonal activation and metabolic dysfunction likely create a milieu that potentiates the pressor effects of triglycerides (24). Firstly, patients with reduced EF often exhibit overactivation of the renin-angiotensin-aldosterone system (RAAS) and the sympathetic nervous system (25), which in itself creates a potent background for elevated blood pressure. Against this backdrop, abnormal TG metabolism may synergize with this neuroendocrine axis, exacerbating vasoconstriction and sodium-water retention. Secondly, the insulin resistance state prevalent in heart failure patients can further exacerbate hypertriglyceridemia by increasing hepatic very-low-density lipoprotein synthesis and reducing peripheral tissue clearance of TG (18). Insulin resistance itself can also activate RAAS and promote oxidative stress, creating a vicious cycle that amplifies the adverse impact of TG on blood pressure. The clinical significance of managing TG in this high-risk population extends beyond its association with hypertension.

### 4.3 Clinical and research implications

Beyond our specific findings regarding hypertension, the broader clinical relevance of TG in high-risk CAD populations is further substantiated by recent evidence. Specifically,our findings resonate strongly with and are powerfully corroborated by a seminal 2025 study (26), which identified triglycerides as a key predictor of extreme cardiovascular risk and event recurrence in CAD patients. This convergence of evidence solidifies the position of TG as a critical residual risk factor in the statin era. Our work extends this paradigm by delineating a specific, independent J-shaped association between TG and the prevalence of hypertension—the most common comorbidity in this population. Thus, while links TG to hard clinical endpoints, our study elucidates its profound impact on a major, pervasive risk factor. Together, these findings compellingly argue that targeted TG management could yield dual benefits: mitigating the risk of major adverse events while simultaneously improving control of pivotal modifiable risk factors like hypertension, especially in high-risk subgroups such as patients with impaired cardiac function (27).

Our results have potential implications for clinical practice. Current guidelines generally recommend maintaining TG levels below 1.7 mmol/L (150 mg/dL) for cardiovascular risk management (28). Our study found that TG levels remain associated with hypertension risk even at higher concentrations (up to 3.86 mmol/L). Therefore, for CAD patients post-PCI, especially those with preserved EF, maintaining TG levels below 3.86 mmol/L might represent a more targeted preliminary management goal. This implies that targeting TG levels below this inflection point, particularly in high-risk subgroups, could represent a tailored therapeutic strategy (29, 30). For patients with EF < 50%, our data strongly suggest that strict control of TG may yield additional benefits for blood pressure management. This approach addresses the unmet need for adjunctive lipid management in high-risk patients, such as those with heart failure, beyond conventional statin therapy (31). This hypothesis urgently needs validation in future prospective interventional studies.

### 4.4 Study strengths and limitations

The strengths of our study include its focus on a well-defined and clinically highly relevant population (CAD undergoing PCI) and being the first to describe the J-shaped relationship between TG and hypertension and the modifying role of EF in this population. However, several limitations must be considered cautiously. First, the retrospective cross-sectional design precludes any causal inference, and the observed associations could be influenced by residual confounding or reverse causality. Second, we could not incorporate data on body mass index or the use of lipid-lowering medications (particularly fibrates and fish oil preparations), which may confound both TG levels and their association with hypertension. Finally, the single-center design and relatively limited sample size may restrict the generalizability of our findings and introduce some instability in subgroup analyses (e.g., the EF < 50% group). Future large-scale, multicenter prospective cohort studies that meticulously collect data on medication use and anthropometric measurements are essential to confirm our observations.

## 5 Conclusion

Among patients undergoing PCI, TG levels exhibited a J-shaped association with hypertension risk, with an inflection point at 3.86 mmol/L. This association was significantly amplified in patients with cardiac dysfunction (EF < 50%). Our findings reveal a strong association between elevated TG and hypertension in this population, particularly when EF is reduced. The identified inflection point (3.86 mmol/L) could serve as a focus for future longitudinal studies to determine if targeted TG control provides clinical benefits, particularly in those with cardiac dysfunction.

## Data Availability

The datasets presented in this article are not readily available because The datasets generated and analyzed during this study are not publicly available due to restrictions in the ethical approval and the informed consent agreements signed by the participants, which explicitly prohibit the public sharing of their raw,anonymized data to protect their privacy and confidentiality. The data are also subject to institutional data protection policies. Requests to access the datasets should be directed to Bo Yu, yubodr@163.com.
